# Conditions and Co-production of Integrated Care for Patients with Multimorbidity

**DOI:** 10.5334/ijic.7648

**Published:** 2024-10-18

**Authors:** Kirstine Skov Benthien, Nina Gøtzsche, Louise Meinertz Jakobsen, Michaela Schiøtz

**Affiliations:** 1 Department of Pulmonary, Hormone, and Endocrine Diseases, Copenhagen University Hospital –Hvidovre, Denmark; 2 Center for Clinical Research and Prevention, Copenhagen University Hospital –Frederiksberg, Denmark

**Keywords:** integrated care, multimorbidity, home nursing, hospital, cross-sectoral, co-production

## Abstract

**Introduction:**

People with multimorbidity can experience fragmented healthcare and burden of treatment and the evidence-base for integrated care in multimorbidity is weak. The aim of this study was to develop a model for integrated care for patients with multimorbidity: The Primary Organization and Relations-Team (PORT).

**Description:**

The PORT prototype was formed using a co-production approach including workshops with healthcare professionals from hospital, general practice and municipalities, and interviews with patients with multimorbidity. The qualitative data were analyzed with systematic text condensation. During the co-production phase, 38 persons were interviewed or participated in workshops. Four themes emerged as central for integrated care for patients with multimorbidity: Information sharing, decision making across sectors, healthcare fragmentation, and patient-centeredness. A prototype aimed at these themes was developed and included continuous information sharing and case management by a joint specialty clinic, a total healthcare plan, and systematic needs assessment.

**Discussion:**

The results and PORT prototype were developed through a comprehensive co-production process and the results and model may be transferred to other healthcare systems that are divided into sectors.

**Conclusion:**

Integrated multimorbidity care may be met through continuous information sharing, case management by a joint specialty clinic, a total healthcare plan, and systematic needs assessment.

## Introduction

With increasing longevity worldwide follows a growing number of persons living with multiple chronic diseases. In a Danish national survey, 33% of the adult population had two or more long-term conditions [1]. In many countries, patients who incur most of the healthcare costs are multimorbid [2,3]. People with multimorbidity are more likely to be frail [4], burdened with symptoms [5], and people with multimorbidity as well as caregivers report reduced health-related quality of life [6,7]. For patients with healthcare providers (HPs) in several specialties and sectors, keeping track of appointments, treatments, and information can be experienced as a treatment burden in addition to the burden of illness [7,8,9]. The number of involved HPs increases with multimorbidity, and healthcare risks becoming fragmented [9,10,11]. Care fragmentation involves several adverse outcomes including redundant work and testing, over-utilization of medicine, medical and communication errors, and adverse hospitalizations [12,13,14,15]. Furthermore, lacking continuity is detrimental to achieving personalized healthcare [11,16]. Justifiably, patients and caregivers express a prominent need for coordinated and patient-centered healthcare [9,17].

Interventions of integrated care (IC) between general practitioners (GP), municipalities and hospital-based specialists intend to remedy fragmented healthcare. Healthcare systems with a strong integration in healthcare services seem to have fewer hospital admissions [18]. IC is characterized by focusing on health needs as opposed to disease and cure, personal relationships as opposed to consultation contacts, and perceiving patients as partners rather than consumers [15]. A retrospective study supports the hypothesis that IC in multimorbidity can reduce hospital admissions [19]. The evidence base for care organization in multimorbidity is weak with mixed results on clinical and patient-reported outcomes and use of healthcare resources [20,21,22]. In summary, there are no evidence-based recommendations for care delivery for multimorbid patients and person-centered IC models are strongly needed [23,24].

The aim of this study was to develop a model for integrated care for patients with multimorbidity: The Primary Organization and Relations-Team (PORT).

### Ethical considerations

The study was conducted in accordance with the Helsinki Declaration. All participants of the development of the intervention prototype provided written, informed consent. Data was collected under the principles of the General Data Protection Regulation and approved by the Data Protection Agency.

## Intervention development

### Design

This study constitutes the first stage of the development and evaluation of a complex intervention as coined by the Medical Research Council: The development [25]. This development stage is succeeded by an ongoing pilot study. The development of the PORT prototype was formed using a co-production approach. Co-production originates from the recognition that if organizations are to deliver successful services, they must understand the needs of their users and engage them closely in the design and delivery of those services [25]. The collaborative process and co-production of the PORT prototype were inspired by different frameworks to support inclusion of a) existing evidence from research and grey literature; b) knowledge and experiences of professionals within hospital, municipality, and general practice; and c) experiences from patients with multimorbidity. The process contains two stages: 1) Stakeholder and evidence consultation and 2) Co-production. O’Cathains framework on how to develop complex interventions was used to guide the actions at each stage [25], and the in-DEPtH framework described by Lo and Karnon [26] guided the combination of research evidence and expert knowledge of local stakeholders and supported condensation and conversion of the multiple data types into prototype components.

### Setting and usual care

**Figure 1:**
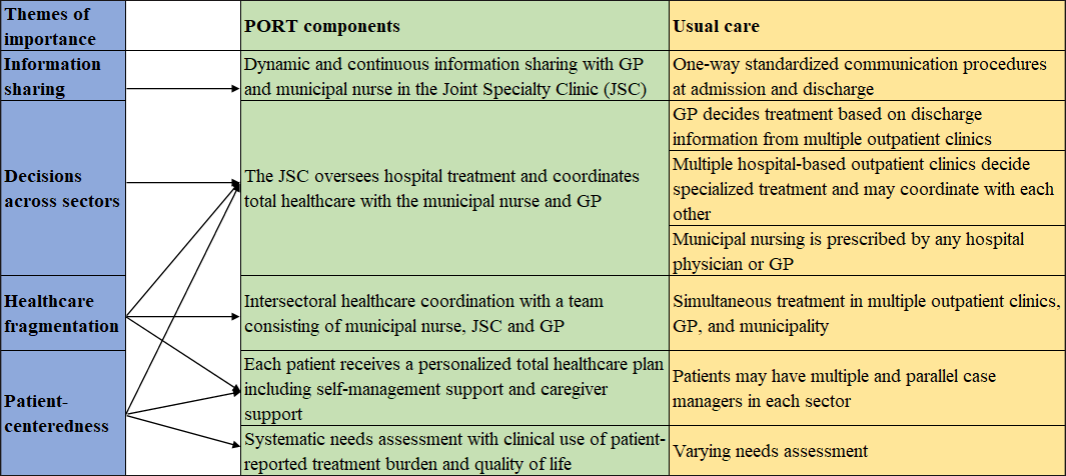
PORT prototype.

The model was developed in the Danish Healthcare System, which is a universal tax financed healthcare system. The Danish healthcare system is divided in three sectors: Hospitals, municipalities, and general practice. The hospitals are managed by five Regions and the 98 municipalities are responsible for providing rehabilitation including 24-hour rehabilitation services, homecare, and home nursing. General practitioners act as gatekeepers to municipal and hospital-based healthcare by referring to hospital-based diagnostics and treatment and manage uncomplex chronic conditions. The study is set in the uptake area of Bispebjerg and Frederiksberg Hospital located in the capital area, Copenhagen. The methods of communication and cooperation in the Danish healthcare system is described in Figure 1 under ‘Usual care’.

### Patient participants

Patient participants were recruited from a hospital and municipal rehabilitation facility. The selection criteria were one hospital admission within six months and two or more of the following chronic diseases: diabetes, heart diseases, connective tissue diseases, pulmonary diseases, kidney diseases, abnormalities of mobility and falls, other urinary and intestinal disorders, cerebrovascular disease, prevalent cancer, and dementia. Patients were selected based on maximum variation on gender, age, and diseases.

### Healthcare professional participants and steering group

Partners from all sectors were represented in the steering group. The steering group consisted of a director from Bispebjerg and Frederiksberg Hospital, the Department head of the Geriatric and Palliative Dept., a GP representative, managers from both municipalities Copenhagen and Frederiksberg, a representative from the patient organization ‘Ældresagen’, and two members of the research team. The steering group appointed HP and managerial workshop participants with a wide range of expertise and perspectives to participate in the co-production workshops because they had key roles and knowledge of multimorbidity and patient trajectories.

### Data collection

The co-production process in this study was based on qualitative data and consists of field notes, audio-recordings and transcripts from formal meetings, individual interviews with patients, and workshops with health care providers. The qualitative data was supplemented by published research evidence and grey literature. The methods used at each stage is described below. In the Supplementary material, Table 1 gives an overview of the activities of the development phase, including their objectives and products.

**Table 1:** Data and participants.


TYPES OF DATA	PARTICIPANTS (N)

Formal start-up meetings (Field notes)	Geriatric and Palliative Department, Bispebjerg and Frederiksberg Hospital Joint Specialty Clinic, Bispebjerg and Frederiksberg Hospital Emergency Department, Bispebjerg and Frederiksberg Hospital Municipality of Copenhagen Municipality of Frederiksberg General practitioners

Individual interviews (Transcriptions and field notes)	Patients with multimorbidity (12)

Workshops within each sector (Transcriptions and field notes)	1.Nurses from with the Geriatric and Palliative Department, Bispebjerg and Frederiksberg Hospital (3) 2.Managers, doctors, nurses, and secretaries from the Joint Specialty Clinic, Bispebjerg and Frederiksberg Hospital (4) 3.Nurses from the Emergency Department, Bispebjerg and Frederiksberg Hospital (2) 4.The municipality of Copenhagen (2) 5.The municipality of Frederiksberg (6) 6.General practitioners in the local area of Frederiksberg Municipality (5) 7.General practitioners in the local area of Copenhagen Municipality (3) + one individual interview

Ad hoc intervention development group meetings (Field notes)	Steering group meeting 1 – GP – JSC management – JCS nurses – Copenhagen Municipality – Frederiksberg municipality – Steering group meeting 2 – Frederiksberg municipality – JSC – GP – Steering group meeting 3

Meetings with steering group (Transcriptions and field notes)	Members of the steering group (8)


#### Stage 1. Stakeholder and evidence consultation

The first step of the co-production process was the designation and involvement of all relevant stakeholders including patients and actors from the different healthcare sectors involved. Formal start-up meetings were held to reach an agreement on the co-production process, the required resources and to ensure the inclusion of all relevant actors for the workshops. Partners from all sectors formed the cross-sectoral steering group and appointed workshop participants. In addition to the international evidence base outlined in the Background section of this article, Danish reports with evaluations of 11 Danish sector-integrated interventions were included as key material.

#### Stage 2. Co-production

Before co-production, the only fixed elements were the partners, who constituted the team in PORT. Workshops were conducted with the HPs separately for each sector as outlined in Table 1 and consisted of two to eight participants and supplementary interviews. The aims of the workshops were to initially explore elements of importance in providing integrated care, which served to secure focus and buy-in for the subsequent topic potential solutions and dream scenarios for integrated care. The workshop script is included in the Supplementary material. Patients were individually interviewed with a semi-structured interview guide aiming to uncover experiences of care and treatment within and across sectors [27,28]. The themes of the interview guide were continuity of care and patient involvement, admissions and transitions, and cooperation with HPs. The interviews began with exploring experiences and then elicited patients’ suggestions for integrated care. The themes are further described in the Supplementary material. Data and participants in workshops and interviews are described in Table 1.

A total of 38 persons participated in 7 workshops for HPs including one interview, that was conducted individually due to scheduling conflicts and 12 patient interviews in addition to the input from the eight members from the steering group. That includes 12 patients, nine GPs, nine hospital staff, and eight municipality staff. Patients’ informal caregivers were contacted for interviews, but they opted out because of caregiver burden. After the analyzing the workshop and interview material, the steering group was presented with the four themes and the according suggestions from the workshops, patient interviews, and literature and then selected the prototype components that were considered possible within the current healthcare system and still potent enough to imply patient benefit. As the following Results section will describe, the potential solutions identified in the early workshops required further development and specification before they were ready for testing. The project was however delayed by COVID-19 priorities which meant that each organization focused on other agendas during development phase. The solutions identified during the early workshops had mixed reception among middle managers post COVID-19 that did not participate in the early workshops. Based on these conditions and the partners’ wishes for the co-production process, the prototype was decided through a series of bilateral meetings to decide on methods, frequency, and content of contact between the JSC and municipalities. At these meetings, which often included middle managers and specialists, the results from workshops and patient interviews were presented. Meetings with individual partners were needed to secure continuous commitment throughout the process and to establish where the partners were willing to adjust their organizations. The meetings were coordinated by the steering group and the process was iterative, building on the Joint Specialty Clinic’s (JSC) treatment approach, adding municipal tasks, and finally general practice tasks.

### Analyses

Interviews and workshops were audio-recorded, and field notes written down immediately after the sessions by the attending researchers. All interviews and workshops were transcribed verbatim by a research assistant. Transcriptions of interviews, workshops and field notes was analyzed using systematic text condensation [29]. The initial condensation into meaning units was performed by two researchers who sorted the meaning units into elements of importance, rationale, existing, and potential solutions regarding sector-integrated care for each stakeholder workshop. The initial condensation of each workshop was approved or elaborated by the participants. Based on the meaning units from the initial condensation, three researchers created and discussed initial themes and two researchers completed the coding with feedback from the other researchers. The themes served as a basis for a joint co-production process across sectors.

### Intervention development results

The results from workshops, interviews, meetings, and key literature are presented in the following. Evidence on IC interventions focuses on single chronic diseases, of which the most promising IC interventions were characterized by having multiple components including personalized care plans and self-management support [20]. Several interventions have been piloted in Denmark, but few of these have been systematically evaluated. An intervention with nurses dually employed in hospital and municipality in another Danish region had promising results with possible reductions in hospital admissions without increasing primary care and municipality services, and an increase in patient satisfaction. However, the intervention was perceived as organizationally challenging and therefore unsustainable [30,31]. The main principle of shared responsibility rather than the organization from that intervention was therefore included in the knowledge base for the PORT development phase.

Results from interviews, workshops and literature covered four themes that are summarized in Table 2. The identified areas of importance were addressed by all stakeholders with differing perspectives.

**Table 2:** Identified areas which impact integration of care.


	PATIENTS	HOSPITAL	MUNICIPALITY	GENERAL PRACTICE	EVIDENCE

Information sharing	Repeatedly new contacts without knowledge	Lacking information and contact information	Lacking information and contact information	Lacking information and contact information	General overview challenging for all parties

Decisions across sectors	Lacking follow-up and contact	Wish for shared decision making	Unclear responsibilities, different views on needs	Unclear responsibilities, wish for shared decision making	Multiple barriers for global decisions

Healthcare fragmentation	Uncertainty about healthcare responsibility	Initiate compensatory interventions	Find coordination tasks challenging	Embrace gate-keeper role but not care coordination	Implementation challenges in primary care

Patient centeredness	Wish for information and a personal relationship with healthcare providers	Challenging to assess supportive needs	Embrace supportive role but is impaired by lacking information	Challenging to assess supportive needs	Treatment burden in addition to disease burden


A further topic identified by some partners was delineation of the target group. Multimorbidity alone was not considered sufficiently specific and further additional criteria such as medical complexity and care needs were proposed. The four themes are elaborated in the following.

#### Information sharing

The workshops revealed that the HP needs for information were frequently unmet in all three healthcare sectors. The traditional focus on transitions between sectors has led to one-way communication products in a structured format where the sender is unsure of the recipients’ information needs and with no direct communication and dialogue. Furthermore, it may be unclear for the recipient who to contact if the received information is insufficient. All HPs desired dynamic information about observations and healthcare provided in other sectors. Several communication products were already in place to share predefined healthcare information in relation to hospital discharge (discharge summaries, care plans, rehabilitation plans), whereas communication tools were not implemented for hospital admission. The standardized communication products were often inadequate as described by a hospital nurse:

*“All the things we line up to make them stable and raise their level of function, it falls apart – especially with these patients. We discharge them […] and we try to communicate with the municipality, but it is mostly in writing and we don’t know how they receive it in the other end. There is not a lot of communication across [sectors] and that is a huge barrier. It is costly in many ways – concretely when patients decline planned diagnostic procedures.”*

The lack of dynamic information sharing is related to the next theme – decision making across healthcare sectors.

#### Decision making across healthcare sectors

GPs as well as hospital staff could identify diagnostic procedures, treatment, and nursing tasks that they wanted to be performed by another sector. Despite their professional assessment of indication and patient need, they had no influence on decisions related to care in other sectors and no direct communication with professionals across sectors with whom they could share their thoughts. In addition, the hHPs expressed that they often lacked knowledge on available competencies and possibilities in the other healthcare sectors. Therefore, potentially beneficial follow-up on treatment changes, observations of health status, and the ensuing preventive measures, were thus not undertaken. Hospital as well as GPs wished for a right to prescribe and decide healthcare tasks to be performed by another sector or hospital-based specialty. One GP expressed it this way:

*“Back in time we had a referral right. Today we have a ‘referral maybe”*.

Simultaneously, all healthcare professionals acknowledged their own lack of insight into capacity, skills, and conditions across sectors. The roles and responsibilities of each sector was unclear.

#### Healthcare fragmentation

Patient healthcare trajectories were considered complex by HPs who struggled getting an overview of numerous concomitant healthcare trajectories with little integration. Case management was deemed crucial but only sporadically performed within the boundaries of each sector. Case management performed by a nurse without physician cooperation was considered inadequate as was case management performed by a physician without nursing support. Case management was considered particularly challenging where patients’ resources are sparse.

The roles and responsibilities of each sector were considered unclear and the unclarity was a source of frustration. GPs are traditionally assigned a gate-keeper role but were aware that this is not synonymous with a care coordinator role. GPs felt more confident in coordinating prescribed medication than coordinating overall healthcare needs and meeting individual patient information needs. The fragmented healthcare system left patients unsure as worded by one patient:

*“To be honest, I don’t feel any kind of coordination at all […]. I have been diagnosed with needing oxygen, but no one told me if that is permanent or something temporary. […]. But if I need to consult someone, is it my GP who is not updated, or where do I go?”*

Patients recounted receiving contradictory information about medical treatment, and the medical treatment was considered complex by HPs as well.

#### Patient centeredness

The patient interviews exposed that numerous HPs were involved in each patient trajectory, which made overall healthcare planning and inclusion of patient preferences inherently difficult. The lack of a shared plan for the patient across sectors was perceived as detrimental to achieving the person-centered approach which was recommended in all HP workshops. The healthcare system was considered inflexible and unable to adapt to patient’s changing needs. Especially, patients with dementia or cognitive impairment were mentioned as vulnerable groups who had unmet needs for coordination and information. One patient articulated the following:

*“I don’t really think they know how to involve [patients]. What they know is the professional, the technical […] They lack pedagogy. And to share and involve people, and that is much, much more important than people think.”*

Another challenging issue was that patients’ multiple self-management tasks may exceed their capacity and the tasks therefore spill over to burdened caregivers or create healthcare inequity in the cases where no caregivers are available.

Patient informants’ need for support in healthcare coordination varied from no unmet needs to complex unmet needs. Some patients had identified a healthcare professional in either sector who they relied on for overview and case management.

Including patients and their preferences in healthcare planning was considered a priority by all partners.

#### PORT prototype

Suggestions for integrated care that emerged during workshops tended to align with a prevalent focus on handovers between healthcare sectors during e.g. hospital discharge rather than reviewing the full and long-term healthcare trajectory of the patients. Other suggestions proposed through the exploration of dream scenarios were major reform changes that would entail dissolvement of the sectors and surpass the competency of a bottom-up project. These suggestions requiring complete healthcare reform include one shared electronic patient record and resource allocation across sectors and managerial rights such as hospital staff assigning municipal nursing tasks or GPs allocating hospital procedures or diagnostics.

The final components chosen by the partners in the steering group were mid-level organizational and procedural changes that are described in Figure 1. Figure 1 presents the themes of importance that emerged during the co-production process, how these themes informed the PORT prototype components, and how the PORT prototype components distinguish themselves from usual care.

The JSC was from the onset considered a key component in the prototype. The JSC combines simultaneous outpatient departments into one outpatient trajectory. The municipal nurse, the GP, and the JSC doctor and nurse will constitute the PORT prototype providers, that oversees the patients’ total healthcare. The JSC will reach out to GP and municipal nurse throughout the treatment trajectory to enable telephone dialogue about the patients’ plan, coordinate tasks and ensure that all HPs work towards the goals set by patients in collaboration with the JSC. At the patients’ first visit to the JSC, patients will be asked open-ended questions about their healthcare priorities and complete a questionnaire about treatment burden [32] that is used for dialogue support. Finally, the question about target group was resolved by applying the referral criteria already in place for the JSC: Two chronic conditions and at least five pharmaceutical drugs.

## Discussion

This study constituted the development phase of the PORT prototype of integrated care for patients with multimorbidity. Four themes were considered important when developing integrated care: ‘Information sharing’, ‘decision making across sectors’, ‘healthcare fragmentation’, and ‘patient-centeredness’. The themes ‘decisions across sectors’, ‘healthcare fragmentation’, and ‘patient-centeredness’ are supported by recurring themes in qualitative studies of patients with multimorbidity including ‘continuity of care’ and ‘involved in decision-making’ [11], whereas ‘Information sharing’ was mainly prompted by HPs.

The PORT prototype components are in line with overarching principles for care in multimorbidity including supporting decision making, patient-centered approaches, self-management support, case management, interdisciplinary team approach, and integrating information technology [33]. As the literature alone would not have provided sufficient detail for operationalizing PORT, the collected empirical data has contributed with deeper insight into how to improve integration of care in the Danish healthcare system. Furthermore, the purpose of exploring themes of importance was more than generating knowledge and the content of the themes was continuously referred to when deciding the content of the PORT prototype. Opinions on care integration may vary between managers and HPs, between sectors, and between HPs and patients. To reach consensus, the results of the workshops were used continuously to inform and justify the decisions. A strength of this study is the inclusion of knowledge extracted from HPs and managers in all sectors, patients, and literature combined into the creation of the PORT prototype.

### Strengths and limitations

The final PORT prototype includes a comprehensive organizational structure within the hospital through the JSC, and a less comprehensive cross-sectoral organizational structure. To accommodate the wishes of the partners, the co-production process began with an open and explorative approach allowing participants to explore dream scenarios. The process of transforming the workshop ideas into a concrete and operationalizable intervention model required several bilateral meetings. Co-production may be time-consuming and sometimes difficult to follow for participants as discussed by Hawkins et al. [34]. Furthermore, intersectoral interventions are bound by legal, financial, and organizational constraints that are not amenable in bottom-up projects. Therefore, future co-production processes about intersectoral interventions could benefit from a frame model with a few core elements that are fixed from the beginning to bridge the gap between dreams and practice.

The workshops and interviews included perspectives and suggestions from patients, HP, specialists, middle managers, and managers, but the decisions in the final PORT prototype relied heavily upon the middle managers inputs. As mentioned earlier, some middle managers did not participate in the initial HP workshops, which mostly included frontline staff, specialists, and some upper management. This probably prolonged the process and may have impeded the willingness to adapt workflow processes to enable intersectoral communication. The role of middle managers is sometimes overlooked but also deemed important in healthcare innovation and implementation [35].

The patient interview results including suggestions for prototype components were not available for the workshops with HPs but performed afterwards, which may have affected the workshop results. However, the patient interview results were shared with the steering group.

### Patient involvement

The patients were interviewed individually which was deemed necessary due to target group frailty and ongoing pandemic measures. Patient interview results including prototype suggestions were shared with the steering group and at all subsequent meetings with partner representatives and thus directly informed the PORT prototype. Furthermore, a representative from the patient organization represented the patients in the steering group.

### Conclusion

Integrated multimorbidity care may be met through continuous information sharing, case management by a joint specialty clinic, a total healthcare plan, and systematic needs assessment.

### Perspectives

The PORT prototype will undergo pilot testing within the next phase of the study. To maximize the acceptability, feasibility, and quality of the intervention and to make sure that it fits with the implementation context, adaptation will continue throughout the pilot phase. The PORT prototype may be transferred and upscaled to other healthcare systems with similar division into sectors or organizational units with separate patient records and finances.
